# Supplementary material for: Antimicrobial Resistance Profiles and Co-Existence of Multiple Antimicrobial Resistance Genes in mcr-Harbouring Colistin-Resistant Enterobacteriaceae Isolates Recovered from Poultry and Poultry Meats in Malaysia
Source: Antibiotics (Basel). 2023 Jun 15;12(6):1060. doi: 10.3390/antibiotics12061060 (PMC10294884; doi:10.3390/antibiotics12061060)
Supplement: Supplementary file 1 [file antibiotics-12-01060-s001.zip › antibiotics-2378717-supplementary.pdf]

Antibiotics-2378717

Supplementary materials

Antimicrobial resistance profiles and co-existence of multiple antimicrobial resistance genes in *mcr*-harbouring colistin-resistant *Enterobacteriaceae* isolates recovered from poultry and poultry meats in Malaysia

Table S1 : List of the primers for AMR gene detection from *Enterobacteriaceae*

Name of antibiotics	Genes	Primers	Sequence	Annealing T_M (°C)	Amplicon size (bp)	Reference
ESBL	*blaTEM*	TEM-F<br>TEM-R	GTC GCC GCA TAC ACT ATT CTC A<br>CGC TCG TCG TTT GGT ATG G	56	258	[34,36]
	blaCTX-M	CTX-M-F<br>CTX-M-R	CGGGAGGCAGACTGGGTGT<br>TCGGCTCGGTACGGTCGA		381	
	blaSHV	SHV-F<br>SHV-R	GCCTTGACCGCTGGGAAAC<br>GGCGTATCCCGCAGATAAAT		319	
Streptomycin	*aadA1*	aadA1-F<br>aadA1-R	TATCCAGCTAAGCGCGAACT<br>ATTTGCCGACTACCTTGGTC	58	447	[37]
Tetracycline	*tetA*	tetA-F<br>tetA-R	GGTTCACTCGAACGACGTCA<br>CTGTCCGACAAGTTGCATGA	56	577	
Gentamicin	*aac-3-IV*	aac-3-IV-F<br>aac-3-IV-R	CTTCAGGATGGCAAGTTGGT<br>TCATCTCGTTCTCCGCTCAT	58	286	
Fosfomycin	*fosA*	fosA-F<br>fosA-R	ATCTGTGGGTCTGCCTGTCGT<br>ATGCCCGCATAGGGCTTCT	56	271	[42–44]
Chloramphenicol	*floR*	floR-F<br>floR-R	ATGACCACCACACGCCCCG<br>AGACGACTGGCGACTTCTCG	55	1213	[38,39]
	catA1	catA1-F<br>catA1-R	AGTTGCTCAATGTACCTATAACC<br>TTGTAATTCATTAAGCATTCTGCC	58	547	[37]
Ciprofloxacin	*aac(6_)-lb*	aac(6_)-lb-F<br>aac(6_)-lb-R	TTGCGATGCTCTATGAGTGGCTA<br>CTCGAATGCCTGGCGTGTTT	55	482	[40,41]

Table S2 : PCR thermal profile for AMR gene detection

Sl No.	PCR steps	***blaTEM, blaCTX-M, blaSHV***	***aadA1***	***tetA***	***catA1, aac-3-IV***	***fosA***	***floR***	***aac(6_)-lb***
1.	Initial denaturation	95 °C for 5 min	95 °C for 3 min,	95 °C for 15 min	95 °C for 15 min	94 °C for 2 min	95 °C for 5 min	94°C for 2 min
2.	Denaturation	95 °C for 30 s	94 °C for 1 min,	94 °C for 30 s	94 °C for 1 min	94 °C for 30 s	94 °C for 30 s	94°C for 45 s
3.	Annealing	56°C for 40 s	58 °C for 90 s	56 °C for 30 s	58 °C for 30 s	56°C for 1 min	55°C 30 sec	55°C for 45 s
4.	Elongation	72°C for 50 s	72 °C for 1 min.	72 °C for 1 min	72 °C for 1 min	72 °C for 1 min	72 °C for 30 sec	72°C for 45 s
5.	Cycle (sl no. 2-4)	30	32	30	30	30	32	32
6.	Final elongation	72°C for 10 min	72 °C for 10 min	72 °C for 10 min	72 °C for 10 min	72 °C for 5 min	72 °C for 5 min	72°C for 10 min
7.	Storage	4°C for ∞	4°C for ∞	4°C for ∞	4°C for ∞	4°C for ∞	4°C for ∞	4°C for ∞
8.	Reference	[34,36]	[37]			[42]	[38,39]	[40,41]

Table S3 : PCR master mixtures in microcentrifuge tube for each isolate

PCR type	Name of antibiotics	Genes	Primers	Primer concentration	For one reaction, total volume = 25 µL				Reference
					Primer	2x master mix	PCR grade water	DNA	
Multiplex	ESBL	*TEM*	TEM-F	10µM	1 µL	12.5 µL	1.5 µL	5 µL	[34,36]
			TEM-R	10µM	1 µL				
		CTX-M	CTX-M-F	10µM	1 µL				
			CTX-M-R	10µM	1 µL				
		SHV	SHV-F	10µM	1 µL				
			SHV-R	10µM	1 µL				
Simplex	Streptomycin	*aadA1*	aadA1-F	10µM	1 µL	12.5 µL	5.5 µL	5 µL	[37]
			aadA1-R	10µM	1 µL				
	Tetracycline	*tetA*	tetA-F	10µM	1 µL	12.5 µL	5.5 µL	5 µL	
			tetA-R	10µM	1 µL				
	Chloramphenicol	*catA1*	catA1-F	10µM	1 µL	12.5 µL	5.5 µL	5 µL	
			catA1-R	10µM	1 µL				
	Gentamicin	*aac-3-IV*	aac-3-IV-F	10µM	1 µL	12.5 µL	5.5 µL	5 µL	
			aac-3-IV-R	10µM	1 µL				
	Fosfomycin	*fosA*	fosA-F	10µM	1 µL	12.5 µL	5.5 µL	5 µL	[43,44]
			fosA-R	10µM	1 µL				
	Chloramphenicol	*floR*	floR-F	10µM	1 µL	12.5 µL	5.5 µL	5 µL	[38,39]
			floR-R	10µM	1 µL				
	Ciprofloxacin	*aac(6_)-lb*	aac(6_)-lb-F	10µM	1 µL	12.5 µL	5.5 µL	5 µL	[40,41]
			aac(6_)-lb-R	10µM	1 µL				

Table S4 : Antimicrobial resistance pattern and MARI of *E. coli* isolates

ID	Sa	AMR patterns	No. Ab	MAR	MAR>0.2	MAR>0.4
E48	CS	CTX, CRO, S, TOB, CN, FOS, TE, CIP, NOR, C, CL	11	0.79	93.75	62.5
E278	M	CTX, CRO, S, TOB, CN, FOS, TE, CIP, NA, C, CL	11	0.79		
E49	CS	S, TOB, CN, TE, CIP, NOR, NA, C, CL	9	0.64		
E172	L	CTX, S, FOS, TE, CIP, NOR, NA, C, CL	9	0.64		
E275	M	CRO, CTX, S, TOB, CN, FOS, TE, C, CL	9	0.64		
E492	M	CTX, CRO, S, CN, FOS, TE, NA, C, CL	9	0.64		
E366	M	CRO, TOB, CN, FOS, TE, NA, C, CL	8	0.57		
E446	M	CTX, S, TOB, CN, FOS, TE, C, CL	8	0.57		
E504	M	CTX, CRO, S, TOB, CN, TE, NA, CL	8	0.57		
K13	CS	TOB, CN, TE, CIP, NOR, NA, CL	7	0.50		
E170	L	S, TE, CIP, NOR, NA, C, CL	7	0.50		
E331	M	S, TE, CIP, NOR, NA, C, CL	7	0.50		
E354	M	CRO, S, TOB, CN, FOS, TE, CL	7	0.50		
E516	M	CTX, S, TE, CIP, NOR, NA, CL	7	0.50		
E298	CS	TOB, CN, TE, CIP, NA, CL	6	0.43		
E314	CS	CTX, CRO, TOB, CN, TE, CL	6	0.43		
E355	M	S, TOB, TE, NA, C, CL	6	0.43		
E494	M	CTX, S, TE, CIP, NA, CL	6	0.43		
E495	M	CTX, CRO, S, CN, TE, CL	6	0.43		
E512	M	S, TE, CIP, NOR, NA, CL	6	0.43		
E297	CS	TOB, CN, TE, C, CL	5	0.36		
E282	M	TE, CIP, C, CL	4	0.29		
E428	M	S, TE, CIP, CL	4	0.29		
E438	M	TE, NOR, NA, CL	4	0.29		
E467	M	S, TE, NA, CL	4	0.29		
E493	M	TOB, TE, CIP, CL	4	0.29		
E505	M	CN, TE, CIP, CL	4	0.29		
E508	M	S, CIP, NOR, CL	4	0.29		
E277	M	TE, NA, CL	3	0.21		
E513	M	TE, NA, CL	3	0.21		
E503	M	TE, CL	2	0.14		
E202	CS	CL	1	0.07		

CTX= Cefotaxime, S= Streptomycin, CIP= Ciprofloxacin, TOB= Tobramycin, CN= Gentamycin, CRO= Ceftriaxone, FOS= Fosfomycin, TE= Tetracycline, NOR= Norfloxacin, NA= Nalidixic acid, C= Chloramphenicol, CL= Colistin, MARI= Multiple antibiotic resistance index, M= meat, CS= cloacal swab, L= litter

Table S5 : Antimicrobial resistance pattern and MARI of *Salmonella* spp. isolates

ID	Samples	AMR pattern	No. Ab	MAR	MAR>0.2 (%)	MAR>0.4 (%)
S283	M	CTX, S, CIP, TOB, CN, CRO, TE. NA, C, CL	10	0.71	68.57	6.25
S242	L	S, CIP, TE, C, CL	5	0.36		
S95	M	CIP, TOB, TE. NA, CL	5	0.36		
S419	M	S, CN, TE, C, CL	5	0.36		
S235	CS	TE, NA, C, CL	4	0.29		
S402	M	CN, TE, C, CL	4	0.29		
S487	M	CN, TE, C, CL	4	0.29		
S232	CS	TE, NA, CL	3	0.21		
S233	CS	TE, NA, CL	3	0.21		
S212	L	CIP, TE, CL	3	0.21		
S98	M	S, TE, CL	3	0.21		
S90	M	TE, CL	2	0.14		
S62	M	CL	1	0.07		
S83	M	CL	1	0.07		
S89	M	CL	1	0.07		
S79	M	CL	1	0.07		

CTX= Cefotaxime, S= Streptomycin, CIP= Ciprofloxacin, TOB= Tobramycin, CN= Gentamycin, CRO= Ceftriaxone, TE= Tetracycline, NA= Nalidixic acid, C= Chloramphenicol, CL= Colistin, MARI= Multiple antibiotic resistance index

Table S6 : Antimicrobial resistance pattern and MARI of *K. pneumoniae* isolates

Strains	Samples	CTX-30	No. ab	MAR	MAR>0.2	MAR>0.4
K54	L	S, CIP, TE, C, CL	5	0.36	83.33	0
K229	CS	CIP, TE, NA, C, CL	5	0.36		
K55	L	CIP, TE, C, CL	4	0.29		
K512	M	S, CIP, TE, CL	4	0.29		
K402	M	CIP, NA, CL	3	0.21		
K218	CS	CL	1	0.07		

S= Streptomycin, CIP= Ciprofloxacin, TE= Tetracycline, C= Chloramphenicol, CL= Colistin, MARI= Multiple antibiotic resistance index

Table S7 : Prevalence of AMR genes in *E. coli* isolated from different sources

Sources	Antibiotic resistance gene profile (%)																			
	^{bla}TEM	*P*-value	*^{bla}SHV*	*P*-value	*^{bla}CTX-M*	*P*-value	*tetA*	*P*-value	*floR*	*P*-value	*catA1*	*P*-value	*aac-3-IV*	*P*-value	*aadA1*	*P*-value	*fosA*	*P*-value	*aac(6')-Ib*	*P*-value
Cloacal swab (n=7)	5 (71.4)	0.227	0	nc	0	nc	5 (71.4)	0.168	3 (42.9)	0.093	0	0.817	4 (57.1)	0.015	3 (42.9)	0.011	1 (14.3)	0.158	3 (42.9)	0.065
Litter (n=2)	1 (50)		0		0		2 (100)		2 (100)		0		1 (50)		2 (100)		0		1 (50)	
Meat (n=23)	8 (34.8)		0		0		10 (43.5)		6 (26.1)		1 (4.3)		2 (8.7)		3 (13)		0		2 (8.7)	
Total (n=32)	14 (43.8)		0		0		17 (53.1)		11 (34.4)		1 (3.1)		7 (21.9)		8 (25)		1 (3.1)		6 (18.8)	

nc= not computed

Table S8 : Prevalence of AMR genes in *Salmonella* spp. isolated from different sources

Sources	Antibiotic resistance gene profile (%)																			
	^{bla}*TEM*	*P*-value	^{bla}*SHV*	*P*-value	^{bla}*CTX-M*	*P*-value	*tetA*	*P*-value	*floR*	*P*-value	*catA1*	*P*-value	*aac-3-IV*	*P*-value	*aadA1*	*P*-value	*fosA*	*P*-value	*aac(6')-Ib*	*P*-value
Cloacal swab (n=3)	0	0.402	0	nc	0	nc	3 (100)	0.148	0	0.402	0	nc	0	0.298	0	0.024	0	0.21	0	0.785
Litter (n=2)	1 (50)		0		0		1 (50)		1 (50)		0		0		1 (50)		1 (50)		0	
Meat (n=11)	4 (36.4)		0		0		4 (36.4)		4 (36.4)		0		4 (36.4)		0		1 (9.1)		1 (9.1)	
Total (n=16)	5 (31.3)		0		0		8 (50)		5 (31.3)		0		4 (25)		1 (6.3)		2 (12.5)		1 (6.3)	

nc= not computed

Table S9 : Prevalence of AMR genes in *K. pneumoniae* isolated from different sources

Sources	Antibiotic resistance gene profile (%)																			
	^{bla}*TEM*	*P*-value	^{bla}*SHV*	*P*-value	^{bla}*CTX-M*	*P*-value	*tetA*	*P*-value	*floR*	*P*-value	*catA1*	*P*-value	*aac-3-IV*	*P*-value	*aadA1*	*P*-value	*fosA*	*P*-value	*aac(6')-Ib*	*P*-value
Cloacal swab (n=2)	0		1 (50)		0		1 (50)		1 (50)		0		0		1 (50)		1 (50)		1 (50)	
Litter (n=2)	0	nc	2 (100)	0.472	0	nc	2 (100)	0.135	2 (100)	0.135	0	nc	0	nc	1 (50)	0.472	1 (50)	1	1 (50)	0.472
Meat (n=2)	0		1 (50)		0		0		0		0		0		0		1 (50)		0	
Total (n=6)	0		4 (66.7)		0		3 (50)		3 (50)		0		0		2 (33.3)		3 (50)		2 (33.3)	

nc= not computed

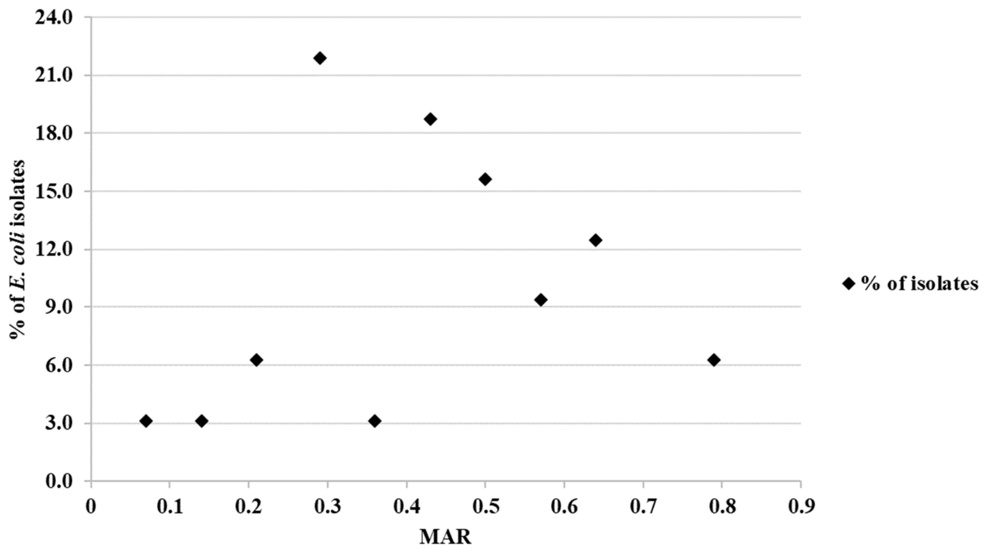

Figure S1 : **MARIs and percentages among colistin-resistant *E. coli*.**

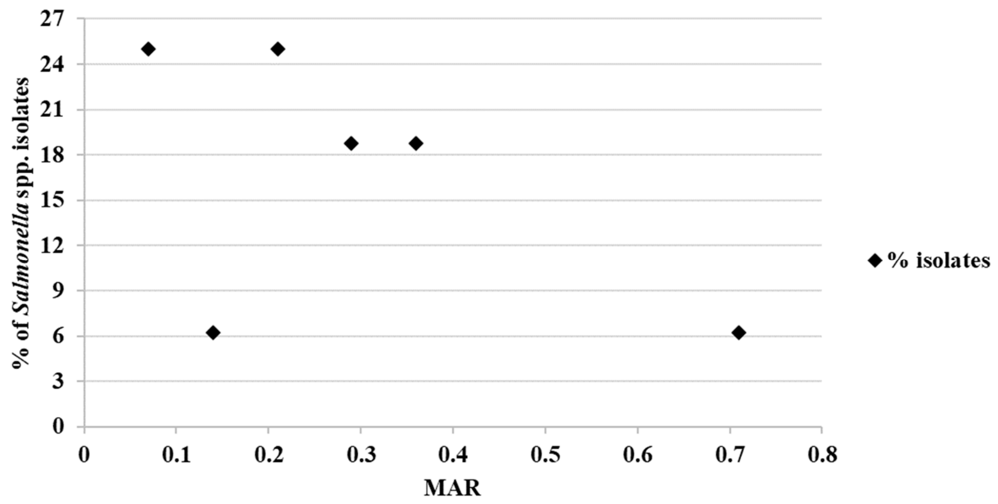

Figure S2 : **MARIs and percentages among colistin-resistant *Salmonella* spp..**

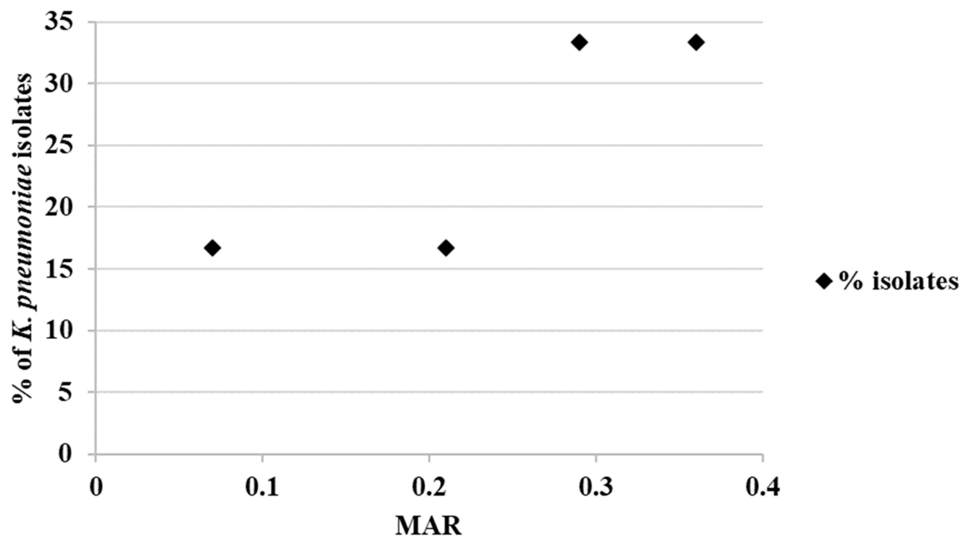

Figure S3 : MARIs and percentages among colistin-resistant *K. pneumoniae*.
